# Systematic Review of Clinical Applications of CAD/CAM Technology for Craniofacial Implants Placement and Manufacturing of Orbital Prostheses

**DOI:** 10.3390/ijerph182111349

**Published:** 2021-10-28

**Authors:** Waqas Tanveer, Angela Ridwan-Pramana, Pedro Molinero-Mourelle, Tymour Forouzanfar

**Affiliations:** 1 Department of Oral and Maxillofacial Surgery, Amsterdam University Medical Center, 1081 HV Amsterdam, The Netherlands; 2 Center for Special Care in Dentistry, Department of Maxillofacial Prosthodontics, Stichting Bijzondere Tandheelkunde, 1081 LA Amsterdam, The Netherlands; a.ridwan@amsterdamumc.nl; 3 Department of Reconstructive Dentistry and Gerodontology, School of Dental Medicine, University of Bern, CHE 3012 Bern, Switzerland; pedro.molineromourelle@zmk.unibe.ch; 4 Department of Oral and Maxillofacial Surgery/Oral Pathology and 3D Innovation Lab, Amsterdam University Medical Center, 1081 HV Amsterdam, The Netherlands; t.forouzanfar@amsterdamumc.nl

**Keywords:** orbital prosthesis, digital planning, digital workflow, craniofacial implants, guided implants surgery

## Abstract

This systematic review was aimed at gathering technical and clinical applications of CAD/CAM technology for the preoperative planning of craniofacial implants placement, designing of molds and substructures and fabrication of orbital prostheses. Following the Preferred Reporting Items for Systematic Reviews and Meta-Analysis (PRISMA) guidelines, an electronic search was executed. Human studies that utilized digital planning systems for the prosthetic rehabilitation of orbital defects were included. A total of 16 studies of 30 clinical cases, which were virtually planned through various digital planning and designing software, were included. The most common preoperative data required for digital planning were CT scans in 15 cases, the 3DSS-STD-II scanning system in 5 cases, an Artec Color 3D scanner in 3 cases and a NextEngine Desktop 3D laser scanner in 2 cases. Meanwhile, the digital designing software were Ease Orbital Implant Planning EOIPlan software in eight cases, Geomagic software in eight cases, Simplant software in four cases and Artec Studio 12 Professional in three cases. Surgical templates were fabricated for 12 cases to place 41 craniofacial implants in the orbital defect area. An image-guided surgical navigation system was utilized for the placement of five orbital implants in two cases. Digital designing and printing systems were reported for the preoperative planning of craniofacial implants placement, designing of molds and substructures and fabrication of orbital prostheses. The studies concluded that the digital planning, designing and fabrication of orbital prostheses reduce the clinical and laboratory times, reduces patient visits and provide a satisfactory outcome; however, technical skills and equipment costs are posing limitations on the use of these digital systems.

## 1. Introduction

Orbital defects can arise from acquired or congenital anomalies. Acquired defects of the orbital location can be seen due to tumors and trauma, while congenital defects appear as the result of developmental anomalies. Exenteration of the eye is one of the most aggressive surgical approaches, which is usually seen after orbital tumor resection. The restoration of an exenteration defect is mostly dependent on the prosthetic options to improve the esthetics and quality of life of these patients [1,2,3,4,5]. With the introduction of endosseous implants, the prosthetic rehabilitation of exenteration orbital defects became less challenging, as they provide improved retention, support and stability of orbital prostheses [3,6]. However, improper planning and placement of these implants can have detrimental consequences on the long-term success and survival of implant-retained prostheses [7,8]. Studies have reported 35–75% success rates upon 3–14 years of follow-up [9,10,11,12,13]. Success rates depend on multiple factors, such as the anatomic location, quality and quantity of bone, systemic health and dose of radiation therapy. In general, the bone in the orbital region is limited, composed of mainly compact bone with little or no marrow, which poses a challenge for the osseointegration of craniofacial implants in this location. Therefore, preoperative planning and intraoperative surgical guides have been increasingly stressed upon for orbital implants placement and the success of orbital implant-retained prostheses [14].

Digital planning systems have brought about revolution in the surgical and prosthetic fields [15]. Computer-aided design and computer-aided manufacturing systems (CAD/CAM) have been in use for implants placement for more than 15 years. By utilizing this 3D technology, customized surgical implant guides can be fabricated, which enables the preoperative planning data to be transferred for intraoperative use in precise implant placement [16]. The preoperative data for digital planning is usually collected either through the use of noncontact three-dimensional imaging such as computed tomography (CT), cone beam-computed tomography (CBCT) and magnetic resonance imagining (MRI) or through various laser scanners. According to Sarment et al. [17], the use of CAD/CAM surgical templates significantly increases the precision and accuracy of dental implant placement when compared with the conventional surgical guides. Furthermore, the use of CAD/CAM technology for craniofacial implants surgery has also been used recently with satisfactory clinical outcomes [18].

More recently, navigation systems have been introduced in craniofacial surgeries. These systems allow surgeons to control the position and movement of instruments with the help of medical images in multiplanar views. Navigation pointer or adapted instruments upon coming into contact with the patient identify the exact location within the radiographic image, giving the operator the ability to simultaneously navigate within the surgical field and virtual anatomical map [19]. With the introduction of navigation systems in the field of dental implantology, two approaches have been used to place implants: dynamic and static navigation. Dynamic navigation works with the help of 3D software, which enables the monitoring of bone drilling and subsequent implant placement in real time throughout the procedure [20,21], while static navigation works through static surgical templates during bone drilling and implant placement.

CAD/CAM technology claims to reduce patient appointments, as well as the clinical and laboratory times of procedures, and reduce the steps of fabrication without compromising the clinical outcome. CAD/CAM systems have been used for the fabrication of surgical templates, models, molds, substructures, customized implants and guided implant surgeries for the prosthetic rehabilitation of orbital defects. The aim of this study is to gather the clinical data to respond to the following question: In patients with orbital defects, what are the technical and clinical applications of CAD/CAM technology for the preoperative planning, designing and manufacturing of orbital prostheses?

## 2. Experimental Section

A systematic review was executed according to the protocol based on all Preferred Reporting Items for Systematic Reviews and Meta-Analyses (PRISMA) [22] for the assessment of the PICO (patients (P), investigation (I), comparison (C) and outcome (O)) question.

Population: Patients with orbital defects.

Intervention: Applications of CAD/CAM technology for preoperative planning and the placement of craniofacial implants.

Comparison: Not applicable.

Outcome: Fabrication of orbital prostheses.

Therefore, the established question was adapted to the PIO question: “In patients with orbital defects (P), what are the technical and clinical applications of CAD/CAM technology for craniofacial implant placement (I) and the manufacturing of orbital prostheses (O)?” This was done while also taking into account that comparison (C) was not applicable in this systematic review.

### 2.1. Search Strategy

The electronic search was conducted by entering a combination of the following terms: (Prostheses AND Planning AND Guide).

Prosthesis: (orbital prostheses OR eye prostheses OR silicone orbital prostheses) AND Planning: (CAD/CAM OR scanning OR digital OR software planning OR navigation OR 3D) AND Guide: (implants OR craniofacial implants OR extraoral implants OR surgical guide OR surgical template OR guided surgery OR printed guide OR navigation system).

### 2.2. Eligibility Criteria

**Figure 1:**
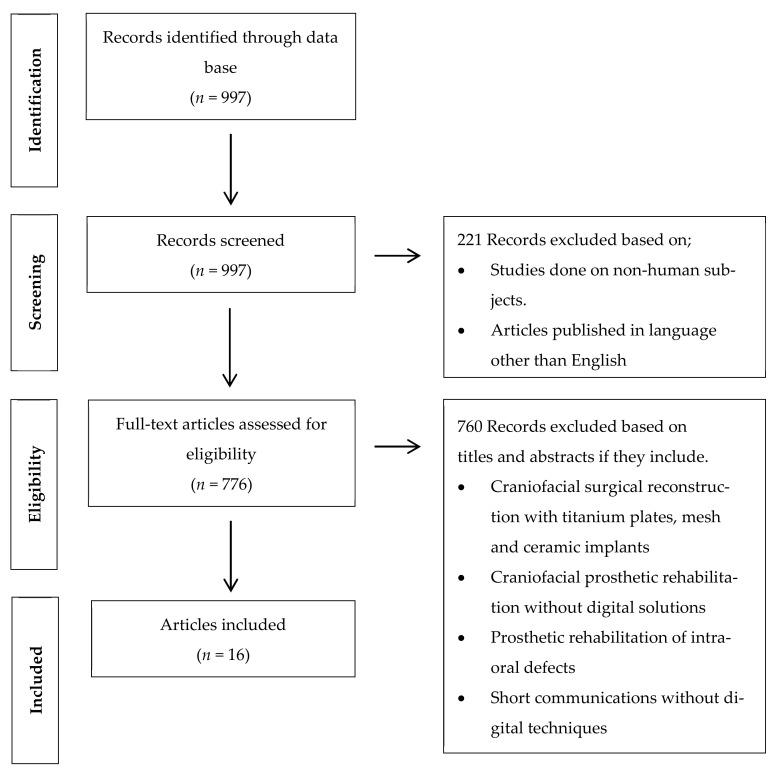
Flow chart of the studies selection process and screening methodology.

Human clinical studies, published in the English language from January 2000 to July 2021, were included in this systematic review. The inclusion criteria involved randomized control trials, cohort studies, case control studies, case series and case reports involving the use of digital planning software for orbital craniofacial implants placement or the fabrication of orbital prostheses. The exclusion criteria were systematic reviews, a finite element analysis (FEA), animal studies, in vitro studies and case reports performed without digital planning software (Figure 1).

### 2.3. Source of Information

An electronic search from January 2000 to July 2021 was conducted in The National Library of Medicine (MEDLINE/PubMed) database.

Moreover, a manual search of the following journals from January 2000 until July 2021 was also performed: *The Journal of Oral Rehabilitation*, *The Journal of Prosthetic Dentistry*, *The Journal of Prosthodontics*, *The International Journal of Prosthodontics*, *The Journal of Prosthodontic Research*, *Clinical Oral Implants Research*, *The Journal of Oral Implantology*, *The International Journal of Oral and Maxillofacial Implants*, *International Journal of Oral and Maxillofacial Surgery*, *Journal of Oral and Maxillofacial Surgery*, *Journal of Cranio-maxillo-facial surgery*, *Journal of Stomatology*, *Oral and Maxillofacial Surgery* and the *British Journal of Oral and Maxillofacial Surgery*, *Implant Dentistry and Clinical Implant Dentistry and Related Research*.

### 2.4. Study Selection

The studies were selected individually by two independent reviewers (W.T. and P.M.M.) through the titles and abstracts of all the identified studies, through the electronic search and further reading individually by the authors. The studies that appeared to fulfill the inclusion criteria, or those studies that had limited data in the title and abstract to reach a final decision, were gathered. Disagreements among the authors were resolved after discussion.

### 2.5. Data Extraction

The data from each included study was extracted according to the designed standard form: Author’s name, year of publication, purpose of digital planning, preoperative data collection, software utilized, printing systems, printing materials, craniofacial implants systems and number of implants placed in each case (Table 1). Contact was made with the authors for possible missing data.

### 2.6. Risk of Bias in Individual Studies

Two independent reviewers (W.T. and P.M.M.) assessed the quality of the included studies. If there was any conflict of agreement regarding any paper, it was further assessed by a third reviewer (A.R.P.). For the assessment step, the critical tools of The Joanna Briggs Institute [23] (JBI) for case series and clinical case reports were used in accordance with the type of included articles. The bias was assessed through a list of 8 questions for case reports and 10 questions for case series, respectively. The questions are specified in Table 2 and Table 3 regarding the risk of bias. Finally, an overall appraisal was made to determine if the risk of bias was low (included), high (excluded) or uncertain (more information needs to be sought). We considered it a high risk of bias if the answer “no” was ≥50%, a low risk of bias if the answer “yes” was ≥50% and an uncertain risk of bias if the “unclear” answer was ≥50%.

## 3. Results

### 3.1. Study Selection

The above-mentioned term was searched through the PubMed database. The flowchart summarized the literature search and selection process in Figure 1. Since the majority of digital advancements for digital planning and printing systems have been seen in the last two decades, the initial search yielded 997 studies with a time filter (January 2000–July 2021). Two hundred and twenty-one studies were excluded by using language (English) and human studies filters. Furthermore, 776 studies were screened on the basis of the titles and abstracts by following the inclusion and exclusion criteria; therefore, an additional 760 studies were excluded based on their rehabilitation techniques and study design (craniofacial reconstruction with titanium plates, ceramic implants and mesh plates, prosthetic rehabilitation of orbital defects without digital solutions and the prosthetic restoration of intraoral defects and ocular implants). After reading full-text papers, a total of 16 studies were included that involved a total of 30 cases planned and executed with digital planning software for the prosthetic rehabilitation of orbital defects (Table 1). Due to the included studies, a quality and data heterogeneity meta-analysis could not be performed.

### 3.2. Study Characteritics

#### 3.2.1. Applications of CAD/CAM Technology for Surgical and Prosthetic Purposes

The included literature mentioned the following purposes for using digital software to virtually plan and execute surgical and prosthetic procedures: mold fabrication for silicone (13 cases), surgical templates (12 cases), the designing of substructures (2 cases), the fabrication of models (2 cases) and dynamic image-guided surgical navigation (2 cases).

#### 3.2.2. Preoperative Record for Digital Planning

Digital preoperative planning required the following records for surgical and prosthetic procedures: CT scans (15 cases), 3D structured light scanning system (3DSS-STD-II; Digital Manu, Shanghai China) (5 cases), Artec Color 3D scanner (Artec 3D, Luxembourg) (3 cases), Laser scanner NextEngine Desktop 3D Scanner (NextEngine, Santa Monica, CA, USA) (2 cases), 3dMDface System (3dMD LLC, Atlanta, Georgia, USA), (2 cases), MRI (1 case), CBCT (1 case), Morpheus 3D Scanner (Morpheus Co., Ltd., Seoul, Korea) (1 case), 3 shape scanner (3 Shape, Copenhagen, Denmark) and Laser scanner VIVID 910 3D noncontact digitizer (Konica Minolta, Osaka, Japan) (1 case).

#### 3.2.3. Preoperative Record for Digital Designing

The digital software utilized by the included studies were Ease Orbital Implant Planning EOIPlan software (Guangdong Provincial Hospital, First Affiliated Hospital, Sun Yat-sen University, Guangzhou, China) (eight cases), Geomagic software (3D Systems, Rock Hill, SC, USA) (eight cases), Simplant software (Dentsply Sirona, York, PA, USA) (four cases), Artec Studio 12 Professional (Artec 3D, Luxembourg) (three cases), Mimics software (Mimics Innovation Suite, Materialise, Leuven, Belgium) (two cases), Digital sculpting software Zbrush software (Pixologic Inc., Los Angeles, CA, USA) (two cases), Brainlab software (Brainlab AG, Munich, Germany) (one case), Rapidform XOS software (INUS Technology, Seoul, Korea) (one case), Autodesk 123D (Autodesk, San Rafael, CA, USA) (one case), Freeform ClayTools software (Freeform, NC, USA) (one case) and NextEngine ScanStudio software (NextEngine, Santa Monica, CA, USA) (one case).

#### 3.2.4. Printing Systems Utilized for Surgical and Prosthetic Phases

Fused deposition modeling (FDM) and stereolithography (SLA) were the two main types of digital printing systems utilized following the digital planning phase for surgical and prosthetic purposes. Fused deposition modeling printers (FDM) Stratasys 400mc (Stratasys, Eden Prairie, MN, USA), ZPrinter^®^ 310 Plus (Z Corp, Cambridge, MA, USA), Zprinter 450 (Z Corp, Cambridge, MA, USA), Ultimaker 3D printer (Ultimaker, Geldermalsen, The Netherlands) and 3D thermojet printer Z510 (Z Corp, Cambridge, MA, USA) were used for seven cases. The printing material used for these cases was acrylonitrile butadiene styrene plastic (ABS). Stereolithography (SLA) printers SLA 7000 (3D Systems, NC, USA), rapid prototyping machine SPS350 (Computer aided technology, Buffalo, NY, USA) and 3D printer EOS P500 (EOS, Krailling, Germany) were used for 10 cases. The printing material used for SLA-based printing systems was polyamide.

#### 3.2.5. Guided Implants Surgery

A total of 44 craniofacial implants were placed in 30 cases after the digital planning and designing of surgical templates. Out of the total 44 implants, 38 implants belonged to the Vistafix implants system (Entific Medical Systems, Goteborg, Sweden), while three implants were used from the Luna implants system (Shinhung Co., Seoul, Korea). Dynamic surgical navigation systems Brainlab (Brainlab AG, Munich, Germany) and Stryker (Stryker Intellect Cranial) were used to guide five implants in two cases.

### 3.3. Risks of Bias in Individual Studies

The JBI criteria was followed to assess the risk of bias of the individual studies. As illustrated by Table 2, the case reports authored by Zhang, X. et al., 2010 [14], Li, S. et al., 2015 [24], Ciocca, L. et al., 2014 [25], Liu, H. et al., 2019 [26], Yoshioka, F. et al., 2010 [27], Sabol, J. et al., 2011 [28], Eo, M.Y. et al., 2020 [29], Huang, Y.H. et al., 2016 [30], Chiu, M. et al., 2017 [31], Choi, K.J. et al., 2016 [32], Ciocca, L. et al., 2010 [33] and Verma, S.N. et al., 2017 [34] showed a low risk of bias. Meanwhile, Table 3 showed that the case series authored by Bi, Y. et al., 2013 [35], Weisson, E.H. et al., 2020 [36], Zhang, X. et al., 2007 [37] and Goh, B.T. 2015 [38] presented a low risk of bias.

**Figure 2:**
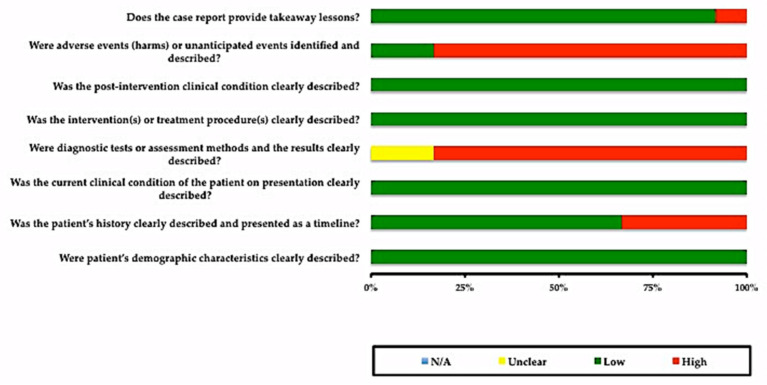
Risk of bias across the included studies for case reports.

In Figure 2, it can be observed that most studies had a low risk of bias ≤ 50%, except for the question, “Were adverse events (harms) or unanticipated events identified and described?”, for which more than 75% of the studies did not mention any adverse event or unanticipated events. For one question, “Were the diagnostic tests or assessment methods and the results clearly described?”, more than 50% of the studies did not clearly mention the diagnostic tests or assessment methods or results of the investigations.

**Figure 3:**
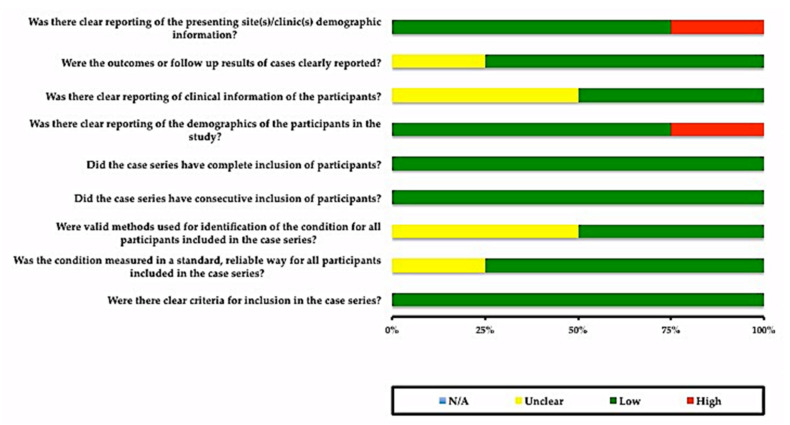
Risk of bias for the case series.

Furthermore, Figure 3 illustrates the risk of bias for four case series studies. Most questions were in favor of a low risk of bias. For two questions, the details were unclear: “Were valid methods used for identification of the condition for all participants included in the case series?” and “Was there clear reporting of clinical information of the participants?”. Furthermore, it was not possible to perform a meta-analysis due to the quality of the included studies, case series and case reports.

## 4. Discussion

Digital planning and designing systems have brought about revolution in dentistry during the last couple of decades. Intraoral implants have been virtually planned and used in computer-guided surgeries since 1997 [39,40]. These digital technology advancements further led to the guided surgeries of craniofacial implants and assisted clinicians and dental technicians in exploring the possibilities of the designing and printing of molds, retentive substructures, customized implants, models, digital wax-ups and prosthesis fabrication [18]. With CAD/CAM application, the surgical procedures became more predicable and reduced the clinical and laboratory times of the surgical and prosthetic steps, reduced the number of patient appointments and provided the patients a chance to virtually visualize the expected outcome before undergoing irreversible procedures [18]. Therefore, the aim of this paper was to gather clinical studies about the various applications of CAD/CAM technology for craniofacial implants placement and the fabrication of orbital prostheses.

Three-dimensional imaging has added an extra dimension to the conventionally available preoperative radiographs with the additional advantage of low radiation doses and detailed information about the bone quantity, bone volume and proximity of adjacent anatomical structures [16,41]. The data from magnetic resonance imaging (MRI), computed tomography (CT) or cone beam-computed tomography (CBCT) can be utilized for preoperative planning by processing through various digital software [42,43]. Therefore, the obtained data from 3D systems facilitates the preoperative planning to guide implants into the most favorable position and angulation without compromising the adjacent critical anatomical structures and prosthetic plan [42,43,44,45]. Among different factors, the slice thickness, pitch, tube current, voltage, image’s slices reconstruction algorithm, slight patient movement and potential artifacts arising from a metal prosthesis can induce errors [46]. The slice thickness has a direct effect on the volume measurements; therefore, it should be kept <1.25 mm [47,48]. In total, four included studies mentioned the slice thickness of CT scans ranging from 1 to 1.25 mm [14,32,37,38]. Meanwhile, the voxel size has influence on the quality, scanning and reconstruction time of CBCT images. A total of two included studies mentioned voxel sizes of 0.3 × 0.3 × 0.3 mm and 0.3 × 0.3 × 2 mm, respectively [30,37].

The integration of laser scans with 3D radiographic imaging introduced the possibility of computer-guided surgeries for implants placement [21,49]. By the incorporation of these two entities in designing software, surgeons and prosthodontists are able to plan computer-guided surgeries in chronological sequence (prosthesis-driven implants placement), from prosthetic planning downwards to the proposed implant position and angulation [50]. In this study, 28 cases were executed by using CT scans, CBCT, MRI and laser scanners; out of which, three cases were planned by the combined use of laser scans and 3D radiographic images for preoperative planning.

Digital planning is dependent on computer-aided design systems, which utilize different software to improve the accuracy of implants placement, designing of molds and models, implant retentive attachments, frameworks, customized implants and provisional and definitive prostheses. In this study, Ease Orbital Implant Planning EOIPlan software and Simplant Pro software were used in a total for 12 cases to plan implant placements in orbital rims. Orbital endosteal implants were guided into the 6:00, 7:00, 9:00, 10:00 and 11:00 O’clock positions for the right orbital rim and 1:00, 2:00, 4:00, 5:00 and 6:00 O’clock positions for the left orbital rim [14,30,37]. Geomagic studio, Artec Studio, Mimics, Zbrush, Rapidform XOS, Autodesk 123D, Freeform ClayTools and NextEngine ScanStudio designing software were used in 16 cases to design molds, models and substructures for silicone packing and orbital prostheses, respectively.

Computer-aided designing (CAD) subsequently leads to computer-aided manufacturing (CAM) to convert the virtual planning and designing into reality by printing models, wax-ups, molds, surgical templates or direct prostheses through the use of fused deposition modeling (FDM) or stereolithography (SLA) based systems [51,52,53,54,55]. In the FDM method, a plastic filament is heated and extruded through an extrusion head on the deposition surface. The extruded plastic gets hard as soon as it is deposited due to the controlled temperature of the air. In this way, successive layers of the deposited material build a physical model. In order to build more complex physical models, accessary extrusion heads are required [53]. FDM technology uses polymers such as polycarbonates, acrylonitrile butyro styrene (ABS) and polysulfones, while the stereolithography method utilizes ultraviolet light to cure the photosensitive resin. Upon each layer of deposition, the ultraviolet light cure, in this way, ultimately builds up the desired complex structure upon successive layers and photopolymerization [53]. SLA-based printing systems use a monomer resin, which, upon photopolymerization, converts into a polymer. FDM printers are mainly used to manufacture models for preoperative planning, molds, provisional crowns and bridges, wax-ups and customized bite registrations, while SLA printers are used to manufacture surgical templates for guided implants surgeries [54]. Each method has its own advantages and disadvantages [54,55]. (Table 4). In the present review, FDM printers were used for seven cases. Acrylonitrile butyro styrene (ABS) was the material used to print molds and surgical templates in all seven cases, while SLA printers were used for 10 cases, and the printing material of choice was polyamide resin.

A total of 44 craniofacial implants were placed in 30 cases after the digital planning and designing of surgical templates. Due to the anatomical morphology of the orbital cavity and better biomechanical support, most studies mentioned three implants for an orbital implant-retained prosthesis [37]. Furthermore, magnet retentive attachments were preferred over clip bar attachments for the retention of orbital prostheses due to the ease of insertion and removal of prostheses and access for hygiene maintenance by the patients [38].

**Figure 4:**
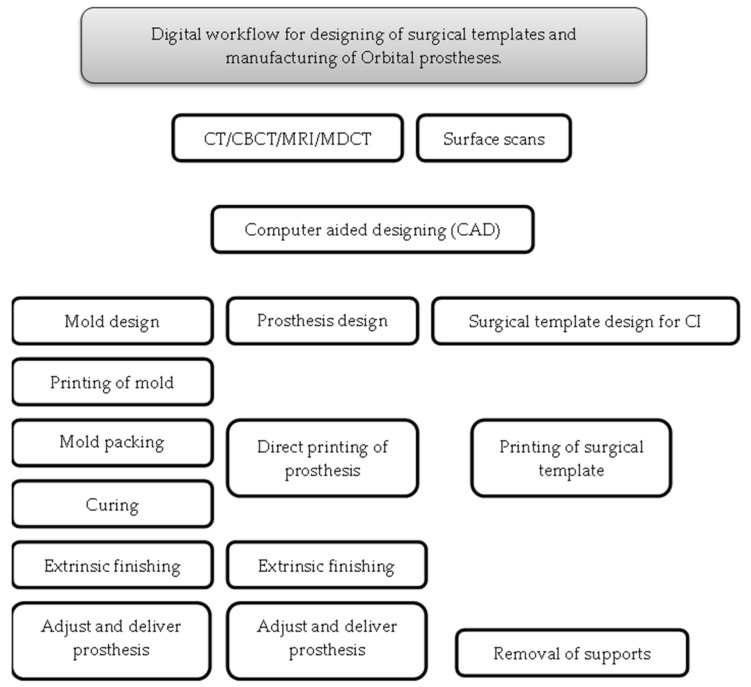
Digital workflow for the designing of surgical templates and manufacturing of orbital prostheses.

The CAD/CAM systems demonstrated the predictable results when rehabilitating patients with orbital defects in numerous studies (Table 5). Digital planning and designing software have enabled the clinical and technical staff to virtually plan cases and discuss the expected outcome with patients prior to invasive surgical procedures. Following the collection of preoperative data, CAD/CAM systems help to virtually plan, design and manufacture molds for silicone prostheses, the direct printing of silicone prostheses and surgical templates for craniofacial implant placement, as illustrated in Figure 4. Additionally, the literature also showed that CAD/CAM systems enable full digital workflows for reasonable times and costs (Table 6). According to Weisson, E.H. et al., 2020 [36], the digital workflow for an orbital prosthesis took 46 h; out of which, 16 h were spent on printing the digital mold, which could be dramatically reduced by newer 3D printers. Sixteen hours were spent on the casting process for silicone at room temperature, which could also be reduced by adjusting the room temperature or using silicone material with less vulcanization time. Bi, Y. et al., 2013 [35] claimed that the whole digital workflow for an orbital prosthesis took 18.5 h from data acquisition to delivery of the prosthesis. Furthermore, a systematic review from Tanveer, W. et al. [18] also mentioned the digital workflow times of different case studies, which were found to be in the range of 12–21 h. However, CAD/CAM systems do pose the limitations of skilled technical staff and expensive equipment hindering their use in many parts of the world. The printing of a direct silicone prosthesis and color matching are other limitations that might be resolved with further digital advancements in the near future.

This systematic review gathered clinical case studies that presented a digital workflow from data acquisition to the designing and printing of models, substructures, molds, surgical templates and provisional prostheses. The available literature has demonstrated that CAD/CAM systems provide predictable outcomes, time- and cost-saving solutions and patient satisfaction. Clinical applications of CAD/CAM systems have shown promising results in this review in terms of orbital implant planning and placement, mold fabrication for silicone packing, the orientation of substructures to retain orbital prostheses, wax-up for quick trial steps and the fabrication of provisional prostheses. Few nonclinical studies [56,57,58] have been conducted to assess the accuracy of CAD/CAM systems in orbital implants placement and the fabrication of orbital prostheses, with varying results (Table 7). However, there are no clinical trials to show the accuracy and precision of these CAD/CAM systems in clinical settings for orbital implants placement and the fabrication of orbital prostheses. Furthermore, the direct printing of silicone orbital prostheses is not yet evident from the literature, which might be due to the limitations of the orientation of ocular prostheses while using the direct printing of silicone. Therefore, future technical developments and clinical trials can be directed to answer these questions.

## 5. Conclusions

CAD/CAM systems have been gaining popularity in pre-surgical and pre-prosthetic planning, designing and printing of implant surgical templates and maxillofacial prostheses. It can be stated that digital planning for the rehabilitation of orbital defects is the most reliable step of the digital workflow as it reduces patient visits, laboratory and clinical time and provides a predictable final outcome. However, the availability of skilled technical staff and equipment costs are still limiting access to digital systems. The direct printing of definitive orbital prostheses is limited by the difficulty in ocular prosthesis orientation within silicone orbital prostheses, the color matching of printed silicone with adjacent skin tones and the marginal thickness. Therefore, further technical advancements are needed to overcome the above-mentioned limitations, while human clinical trials would help to determine the accuracy and precision of these digital systems for craniofacial implant placement and the fabrication of orbital prostheses.

## Figures and Tables

**Table 1:** Digital planning for the prosthetic rehabilitation of orbital defects.

Publications	No. of Cases	Purpose of Software Planning	Pre-op Data for Digital Planning	Software	Printer/Miller	Printing Materials	Navigation System (Yes/No)	Location & No. of Implants	Implants System
Zhang, X. et al., 2010 [14]	1	Surgical template for implants placement	CT	Ease Orbital Implant Planning Software (EOIPlan) (Guangdong Provincial Hospital, First Affiliated Hospital, Sun Yat-sen University, Guangzhou, China) ^1^	Rapid prototype; FDM 400mc, Stratasys (Stratasys, Eden Prairie, MN, USA)	NM	No	Orbital bone rim; 3 implants	Vistafix implant system (Entific Medical Systems)
Li, S. et al., 2015 [24]	1	Simulation for orbital prosthesis and planning for orbital implants	CT	Ease orbital implant planning software (EOIPlan) (Guangdong Provincial Hospital, First Affiliated Hospital, Sun Yat-sen University, Guangzhou, China) ^1^	NM	NM	No	Virtual planning	Virtual planning
Ciocca, L. et al., 2014 [25]	1	Fabrication of Orbital prosthesis substructure and mold	MRI, Laser scan (Desktop NextEngine; NextEngine, Santa Monica, CA, USA)	Freeform Modeling Plus software (3D Systems Inc., Rock Hill, SC, USA) ^10^	RP machine (Phantom Desktop Haptic device, ClayTools system; Sensable, Wilmington, MA, USA)	Polyamide resin	No	No implants	No implants
Liu, H. et al., 2019 [26]	1	Fabrication of mold for orbital prosthesis	3dMDface System (3dMD LLC, Atlanta, Georgia, USA), 3 shape scanner (3 Shape scanner, Copenhagen, Denmark)	Geomagic studio 2014; (3D Systems Inc., Rock Hill, SC, USA) ^2^	3D printer EOS 500 (EOS, Krailling, Germany)	polyamide (PA2200; EOS) (EOS, Krailling, Germany)	No	No implants	No implants
Yoshioka, F. et al., 2010 [27]	1	Fabrication of mold	VIVID 910 3D noncontact digitizer (Konica Minolta, Osaka, Japan)	Softwares; Mimics (Mimics Innovation Suite, Materialise, Leuven, Belgium) ^5^ and Magics, ^2^ (3D Systems Inc., Rock Hill, SC, USA)	3D thermojet printer (Z510, Z Corp, Cambridge, MA, USA)	Hybrid plaster: ZP 150, (Z510, Z Corp, Cambridge, MA, USA)	No	No implant	No implant
Sabol, J. et al., 2011 [28]	1	Fabrication of 3D model for orbital prosthesis	3dMDface System (3dMD LLC, Atlanta, Georgia, USA)	Magics version 12.01 (3D Systems Inc., Rock Hill, SC, USA) ^2^	Zprinter 450 (Z Corp, Cambridge, MA, USA), SLA 7000, a Stereolithography Apparatus (3D Systems, Rock Hill, SC, USA).	zp130 powder (3D Systems Inc., Rock Hill, SC, USA)	No	No implants	No implants
Eo, M.Y. et al., 2020 [29]	1	Fabrication of master cast	CT scan, Morpheus 3D Scanner^®^ (Morpheus Co., Ltd., Seoul, Korea)	ZBrush^®^ software (Pixologic Inc., Los Angeles, CA, USA) ^6^	NM	NM	No	3 implants in the lateral orbital rim of the zygoma	4.0-mm-diameter and 7.0-mm-long Luna^®^ implants (Shinhung Co., Seoul, Korea)
Huang, Y.H. et al., 2016 [30]	1	Surgical template for implants placement, model of patient’s skull	CBCT, Next Generation 17-19, Imaging Sciences International, Hat eld, PA, USA)	Mimics Materialise (Mimics Innovation Suite, Materialise, Leuven, Belgium) ^5^	uPrint SE Plus 3D Printer (Stratasys, Eden Prairie, MN, USA), ZPrinter (ZPrinter 310 Plus (Z Corp, Cambridge, MA, USA))	Acrylonitrile butadiene styrene plastic (ABS)	No	5 implants (four 3 mm and one 4 mm) in the right orbital rim. (Lateral orbital rim location)	Craniofacial implants (Vista x^®^ Prior Generation, Cochlear^TM^, Sydney, NSW, Australia)
Chiu, M. et al., 2017 [31]	1	Fabrication of mold	Digital photographs of patient	Autodesk 123D (Autodesk, San Rafael, CA, USA) ^9^, Digital sculpting software (Z-Brush, Pixologic Inc., Los Angeles, CA, USA) ^6^	NM	NM	No	No	No
Choi, K.J. et al., 2016 [32]	1	Image guided placement of implants by utilizing navigation probe	CT scan	Brainlab (Brainlab AG, Munich, Germany) ^7^	No	No	Yes	2 implants in right superior orbital rim.	Vistafix system (Entific Medical Systems, Goteborg, Sweden)
Ciocca, L. et al., 2010 [33]	1	Fabrication of mold and substructure	Laser scan; NextEngine Desktop 3D Scanner (NextEngine, Santa Monica, CA, USA)	NextEngine ScanStudio software (NextEngine, Santa Monica, CA, USA) ^11^, Rapidform XOS software (INUS Technology, Seoul, Korea) ^8^	Stratasys 3D printer (Stratasys, Eden Prairie, MN, USA)	Acrylonitrile butadiene styrene plastic (ABS)	No	No	No
Verma, S.N. et al., 2017 [34]	1	Image guided surgical navigation for orbital implants placement	CT scan	Stryker, Intellect Cranial, (Stryker Navigation system, Kalamazoo, MI, USA) ^12^	No	No	Yes	3 implants in right superior orbital rim.	NM
Bi, Y. et al., 2013 [35]	5	Fabrication of mold for orbital prosthesis	3D structured light scanning system (3DSS-STD-II; Digital Manu, Shanghai China)	Geomagic Studio 11.0 (3D Systems Inc., Rock Hill, SC, USA) ^2^	Stereo Lithography (SLA) rapid prototyping machine (SPS350; Hengtong Inc, Buffalo, NY, USA)	Resin	No	No implants	No implants
Weisson, E.H. et al., 2020 [36]	3	Fabrication of mold	Artec Color 3D scanner (Artec 3D, Luxembourg city, Luxembourg), colorimeter (E-Skin by Spectro-Match Ltd., Bath, UK)	Artec Studio 12 Professional (Artec 3D, Luxembourg) ^4^, Geomagic Studio 12 (3D Systems Inc., Rock Hill, SC, USA) ^2^	Ultimaker 3D printer (Ultimaker, Geldermalsen, The Netherlands)	Acrylonitrile butadiene styrene (ABS)	No	No	No
Zhang, X. et al., 2007 [37]	6	Surgical template for implants placement	CT scan,	Ease Orbital Implant Planning System (EOIPlan) (Guangdong Provincial Hospital, First Affiliated Hospital, Sun Yat-sen University, Guangzhou, China) ^1^	Rapid prototype; FDM 400mc, Stratasys (Stratasys, Eden Prairie, MN, USA)	NM	No	17 implants in total were placed in supraorbital and zygomatic regions	Craniofacial Vistafix implants system (Entific Medical Systems, Goteborg, Sweden)
Goh, B.T. 2015 [38]	4	Surgical templates for implants placement	CT scan	Simplant Pro CMF module (Dentsply Sirona, York, PA, USA) ^3^	Stereolithographic printer (Materialise, Leuven, Belgium)	NM	No	Total of 11 implants were placed in orbital rims of 4 patients.	Vistafix implants (Entific Medical Systems)

Abbreviations: CT: computed tomography, CBCT: cone beam-computed tomography, Pre-op: preoperative, Post-op: post-operative and NM: not mentioned. ^1^ Ease Orbital Implant Planning System (Guangdong Provincial Hospital, First Affiliated Hospital, Sun Yat-sen University, Guangzhou, China), ^2^ Geomagic Studio software (3D Systems Inc., Rock Hill, SC, USA), ^3^ Simplant Pro CMF module (Dentsply Sirona, York, PA, USA), ^4^ Artec Studio 12 Professional (Artec 3D, Luxembourg city, Luxembourg). ^5^ Mimics Materialise (Mimics Innovation Suite, Materialise, Leuven, Belgium). ^6^ Digital sculpting software (Z-Brush, Pixologic Inc., Los Angeles, CA, USA). ^7^ Brainlab software (Brainlab AG, Munich, Germany). ^8^ Rapidform XOS software (INUS Technology, Seoul, Korea). ^9^ Autodesk 123D (Autodesk, San Rafael, Santa Monica, CA, USA). ^10^ Freeform Modeling Plus software (3D Systems Inc., Rock Hill, SC, USA). ^11^ NextEngine ScanStudio software (NextEngine, Santa Monica, CA, USA). ^12^ Stryker, Intellect Cranial, (Stryker Navigation system, Kalamazoo, MI, USA).

**Table 2:** Risk of bias for the case reports.

Assessment	Author and Year						
	Zhang, X. et al., 2010 [14]	Li, S. et al., 2015 [24]	Ciocca, L. et al., 2014 [25]	Liu, H. et al., 2019 [26]	Yoshioka, F. et al., 2010 [27]	Sabol, J. et al., 2011 [28]	Eo, M.Y. et al., 2020 [29]
Were patient’s demographic characteristics clearly described?	Yes	Yes	Yes	Yes	Yes	Yes	Yes
Was the patient’s history clearly described and presented as a timeline?	Yes	No	No	No	Yes	Yes	Yes
Was the current clinical condition of the patient on presentation clearly described?	Yes	Yes	Yes	Yes	Yes	Yes	Yes
Were diagnostic tests or assessment methods and the results clearly described?	No	No	No	No	No	No	Unclear
Was the intervention(s) or treatment procedure(s) clearly described?	Yes	Yes	Yes	Yes	Yes	Yes	Yes
Was the post-intervention clinical condition clearly described?	Yes	Yes	Yes	Yes	Yes	Yes	Yes
Were adverse events (harms) or unanticipated events identified and described?	No	No	No	No	No	Yes	No
Does the case report provide takeaway lessons?	Yes	Yes	Yes	Yes	Yes	Yes	Yes
Overall appraisal	Included	Included	Included	Included	Included	Included	Included
	**Huang, Y.H. et al., 2016 [30]**	**Chiu, M. et al., 2017 [31]**	**Choi, K.J. et al., 2016 [32]**	**Ciocca, L. et al., 2010 [33]**		**Verma, S. N. et al., 2017** [34]	
Were patient’s demographic characteristics clearly described?	Yes	Yes	Yes	Yes		Yes	
Was the patient’s history clearly described and presented as a timeline?	Yes	Yes	Yes	Yes		No	
Was the current clinical condition of the patient on presentation clearly described?	Yes	Yes	Yes	Yes		Yes	
Were diagnostic tests or assessment methods and the results clearly described?	No	No	No	Unclear		No	
Was the intervention(s) or treatment procedure(s) clearly described?	Yes	Yes	Yes	Yes		Yes	
Was the post-intervention clinical condition clearly described?	Yes	Yes	Yes	Yes		Yes	
Were adverse events (harms) or unanticipated events identified and described?	No	No	Yes	No		No	
Does the case report provide takeaway lessons?	Yes	Yes	Yes	No		Yes	
Overall appraisal	Included	Included	Included	Included		Included	

**Table 3:** Risk of bias for the case series.

Assessment	Author and Year			
	Bi, Y. et al., 2013 [35]	Weisson, E.H. et al., 2020 [36]	Zhang, X. et al., 2007 [37]	Goh, B.T. 2015 [38]
Were there clear criteria for inclusion in the case series?	Yes	Yes	Yes	Yes
Was the condition measured in a standard, reliable way for all participants included in the case series?	Yes	Unclear	Yes	Yes
Were valid methods used for identification of the condition for all participants included in the case series?	Unclear	Yes	Unclear	Yes
Did the case series have consecutive inclusion of participants?	Yes	Yes	Yes	Yes
Did the case series have complete inclusion of participants?	Yes	Yes	Yes	Yes
Was there clear reporting of the demographics of the participants in the study?	Yes	Yes	Yes	Yes
Was there clear reporting of clinical information of the participants?	Unclear	Yes	Unclear	Yes
Were the outcomes or follow up results of cases clearly reported?	Yes	Yes	Unclear	Yes
Was there clear reporting of the presenting site(s)/clinic(s) demographic information?	Yes	Yes	Yes	Yes
Overall appraisal	Included	Included	Included	Included

**Table 4:** Comparison between stereolithography (SLA) and fused deposition modeling and their applications (FDM) [17,51,52,53,54,55].

	Stereolithography (SLA)	Fused Deposition Modeling (FDM)
Materials for printing	Resins	Acrylonitrile Butyro Styrene (ABS) Calcium phosphate based material Polycarbonates Polyphenylsulfones Nylon
Advantages	Short working time High product resolution No deformation More efficient for complex models	Relatively cheaper Post-processing no chemicals
Disadvantages	Limited mechanical strength (mechanical strength depends on the viscosity of resin used) Irritant Relatively expensive	Long working time Low product resolution Limited shape complexity Gradual deformation
Thickness of layer	0.05–0.015 mm	0.5–0.127 mm
Possible applications for facial prosthesis fabrication	Surgical templates for computer guided surgeries Retentive substructures	Models after surface scans Molds Wax-ups Provisional prosthesis

**Table 5:** Enlisted are the clinical outcomes, recommendation and limitations mentioned in the included clinical studies.

Included Articles	Outcome	Recommendations	Limitations
Zhang, X. et al., 2010 [14]	According to the authors, the digital surgical template was precisely designed for specific surface topography or orbital bone, therefore the template was extremely stable and no external fixation was required.	Magnetic retention was recommended for orbital prosthesis due to the ease of placement and removal without compromising retention of prosthesis.	-
Li, S. et al., 2015 [24]	The biggest measurement error was less than 0.3 mm and the variance was less than 0.03. The system provided the simulated rehabilitation images, which were helpful in preoperative planning.	According to surgical team this error was claimed to be acceptable and satisfies the clinical requirements regarding orbital implants placement.	-
Ciocca, L. et al., 2014 [25]	Through this technique it was possible to reduce the thickness (≤1 mm) within 1.5 cm area along the margins with progressive increase in thickness in bulk area of silicone. Furthermore, this technique enabled the connection of eyeglasses and prosthesis with the help of digitally designed substructure, which enhanced the retention.	-	According to the authors, the availability of CAD skilled technician can be limited. Furthermore, Closure of oculo-facial defect by the help of myocutaneous flap could be a prosthetic limitation due to the degree of difficulty in adapting the thin margins of prosthesis to mobile surface of flap during the movement of cheek.
Liu, H. et al., 2019 [26]	This technique saved time and labor compared with conventional method.	The use of intraoral scanner can reproduce skin surface texture therefore authors claimed that additional manual sculpturing is not necessary.	The used technique is applicable to unilateral orbital defects.
Yoshioka, F. et al., 2010 [27]	The photo mapping function of mimics enabled confirmation of the external profile and position of pupil on designed model, which was not possible to locate accurately through CT scan or convectional impression as patient need to close the eyes.	This report presented non-contact laser scanning method, which was clammed to be safer than CT scan as the patient will not be exposed to unnecessary radiation dose	
Sabol, J. et al., 2011 [28]	3D photography technique provided an STL model and 3D printed model for fabrication of orbital prosthesis. There were no ultimate differences in the fit of orbital prostheses fabricated on these models.	Authors recommended the fabrication of intraoral prosthesis before the orbital prosthesis, as the contours of skin should be stable before capturing the image.	The limitations stated were the high cost of CAD/CAM systems and inability to match the color with adjacent skin.
Eo, M.Y. et al., 2020 [29]	The combination of 3D scanning with digital planning and reconstruction resulted in accurate orbital prosthesis in short time. The patient had reported excellent satisfaction for esthetics and stability of orbital prosthesis. The ability to reproduce major mold resulted in accurate silicone morphology.	-	Authors highlighted the limitation of silicone bonding with metal components, using plastic clay resin.
Huang, Y.H. et al., 2016 [30]	The surgical guide obtained after digital planning was found to resist any movement upon seating, which indicated accurate fitting between the bone and surgical guide. Furthermore, surgical guide reduced the operating time.	-	According to the authors, time and cost spent for designing and production of surgical template was favorable but more detailed time and cost comparison will give better understanding of cost effectiveness of surgical templates.
Chiu, M. et al., 2017 [31]	The presented technique utilized digital camera instead of CT/MRI, which reduced the cost of data acquisition	3D photography was recommended in place of CT scans as it reduced the unnecessary radiation dose exposure and gives color images which are important for maxillofacial prosthesis	The limitations expressed by authors were the need of specialized skills and the cost of digital printers, which might not be available in rural location. Furthermore, it was highlighted that 3D printing of medical grade silicone is not available
Choi, K.J. et al., 2016 [32]	Image guided system was used for 3 cases. In patients 1 and 2, implants were real time guided even after extensive soft tissue debridement. For patient 3, alternative implant site with adequate amount of bone was successfully identified and implant was guided in place through image-guided system.	Authors suggested indications of image guided implants placement in head and neck cancer patients, which present altered anatomy, inadequate amount of bone and prior free flap reconstruction.	-
Ciocca, L. et al., 2010 [33]	The CAD/CAM technique along with “Ear & Nose Library” dictated the fabrication of provisional orbital prosthesis, which helped in immediate recovery following ablative cancer surgery and improved the quality of life of patient.	Immediate recovery from this provisional prosthesis is useful after ablative surgery. Titanium framework was recommended to support the facial prosthesis instead of ABS framework to reduce the bulk and improve the rigidity of attachment.	-
Verma, S.N. et al., 2017 [34]	This technique saved approximately 12 h of laboratory time, which is normally spent on designing surgical template. Eliminated two clinical preoperative visits of patient. The virtual planning allowed the surgeon to plan the incision and implant emergence site according to prosthetic boundaries.	While treating the unilateral defect, the implants location and prosthetic design are decided on the basis of mirrored image data from non-affected side. Therefore with all virtual planning, preoperative impression is not required.	Limitation of this specific technique was the inability to estimate the trajectory of registered drill in contra-angle hand-piece during implants osteotomy when utilizing this navigation platform. As the contra-angle hand-piece might be needed in anatomical location with limited space such as while orbital implants placement.
Bi, Y. et al., 2013 [35]	The ocular defect was accurately restored. The structure of eyelid and the wrinkles were clear. The size, marginal adaptation and contour were acceptable. The digital planning and designing saved the time and reduced the visits of patients.	Following silicone vulcanization, the lower pieces of mold should be separated first, and ocular resin models should be carefully removed from prosthesis after they separate from upper pieces of mold. This sequence will prevent damage to silicone prosthesis.	-
Weisson, E.H. et al., 2020 [36]	The data set allowed fast and accurate sculpturing to refine the prosthesis edge-skin interface without the need of patient physically present.	This technique reduces the cost and fabrication time of prosthesis in the case, if it is damaged or lost.	The 3D printer utilized in this study took 20 h to print the mold, which according to authors, can be reduced by industrial type 3D printers.
Zhang, X. et al., 2007 [37]	The implants were successfully and precisely placed according to digital planning. No complications were encountered. Surgical time had reduced as compared to conventional methods. Orbital prosthesis had fit over the implants and patients were satisfied from final outcome.	Improvement in software is needed, to enable mirroring of healthy eye on the defect side, which is important for orientation during planning.	The number of cases was too small for statistical analysis to check the accuracy of this system.
Goh, B.T. 2015 [38]	The stereolithographic surgical template served to transfer individualized plan to operating room for orbital implants placement. During surgery, templates fitted well on orbital rim. There was no need to shift the implant position from originally planned position. Surgery was more predictable and less dependent on surgical skills.	Magnetic retention was recommended as it offers adequate retention, ease of orbital prosthesis placement and better access for hygiene maintenance.	According to authors, long-term survival of implants in orbital defect area was unpredictable due to poor bone quality and radiation dose.

**Table 6:** Estimated time for the designing and manufacturing of orbital prosthesis through digital workflow.

Weisson, E.H. et al., 2020 [36]		Bi, Y. et al., 2013 [35]	
Procedures	Estimated Time (hour)	Procedures	Estimated Time (hour)
Facial topography mapping	1 h	Data acquisition	5 h
Digital design and printing of mold	26 h	Fabrication of prosthesis	
Manufacturing of prosthesis	17 h	Finishing and delivery	
Finishing and delivery	2 h	Digital design and fabrication	13.5 h
Total time	46 h	Total time	18.5 h

**Table 7:** Nonclinical studies that assessed the accuracies of digital systems for orbital implants placement and the fabrication of orbital prosthesis.

Publications	Purpose of Software Planning	Pre-Op Data for Digital Planning	Software	Printer/Miller	Printing Materials	Outcome	Limitations
Bockey, S. et al., 2018 [56]	To Assess, if the method of digital designing of orbital models for orbital prostheses is suitable for patients? Comparison was made among 4 groups; mirror eye, CAD prosthesis, cut out defect area and manually designed prosthesis	3D surface scans of 32 patients were captured through fringe projection method. Measuring accuracy of high resolution is 0.3 mm	3D software; pVision3D, Rhinoceros (Robert McNeel & Associates, version 4.0)	3D printer (OBJET EDEN 260V, Stratasys Ltd., Eden Prairie, USA). With accuracy of 600 dpi lateral and 1600 dpi axial.	VeroDent MED-670 and VeroDent Plus MED-690.	Mirrored eye had the lowest asymmetry index (AI) while the manually designed orbital prosthesis had highest asymmetry index. The deviation of mean values between these groups was 0.33. The results indicated that the use of this technique can help to increase the facial symmetry of prosthesis and can further assist Anaplastologists in technical procedures, as the mirrored eye is the direct template of prosthesis, which could be adjusted manually.	In this study, the question of appropriate implant location and subsequent reconstruction heights were not included. It was not possible to record the position of abutments due to undercuts. Furthermore, this method of reconstruction is only suitable for paired structures, such as eyes, ears.
Marafon, P.G. et al., 2010 [57]	To determine the dimensional accuracy of orbital prostheses manufactured through CAD/CAM system by utilizing CT scan.	CT scan	InVesalius Software (CTI-Information Technology Center), Magics Software (Magics X SP2 v.1.1.17, Materialise), Rhinoceros Software v. 4.0 (Robert McNeel & Associates), SolidWorks Software v. 2008 (Dassault Systèmes)	Selective laser sintering (SLA) (Sinterstation 2000, 3D Systems)	Polyamide (Robotec)	There was no significant difference between the position of landmarks on the prosthesis and the landmarks on the face, indicating no significant displacement of orbital prosthesis in transverse or oblique directions. Furthermore no significant difference between the measurements of the cast and on the orbital prosthesis, thus indicating that the dimensions of orbital prosthesis were stable in transverse, oblique and vertical directions. The dimensional accuracy of orbital prosthesis suggested that CAD/CAM system maybe suitable for clinical use.	-
Dings, J.P.J. et al., 2019 [58]	To determine the accuracy of guided implants placement by using CAD/CAM system to design skin supported digital surgical template	CBCT	Maxilim software (Medicim NV, Mechelen, Belgium), Procera System (NobelGuide; Nobel Biocare, Goteborg, Sweden)	NM	Biocompatible resin	One hundred and thirty-six craniofacial Branemark MK III implants were placed in 10 cadaver heads. Out of total 136 implants, 57 implants were placed in orbital region. The use of fixation pins showed higher mean deviation at implants shoulder, angle and depth when compared to non-fixated surgical templates. Surgical templates without fixation pins showed non-significant difference in angular deviation.	All cadaver heads were edentulous therefore there were no ideal fixed reference points to design the hard tissue supported surgical templates.

Abbreviations: CT: computed tomography, CBCT: cone beam-computed tomography, Pre-op: preoperative, Post-op: post-operative and NM: not mentioned.

## Data Availability

Not applicable for this systematic review.
